# RBE and OER within the spread-out Bragg peak for proton beam therapy: *in vitro* study at the Proton Medical Research Center at the University of Tsukuba

**DOI:** 10.1093/jrr/rru043

**Published:** 2014-05-29

**Authors:** Ayae Kanemoto, Ryoichi Hirayama, Takashi Moritake, Yoshiya Furusawa, Lue Sun, Takeji Sakae, Akihiro Kuno, Toshiyuki Terunuma, Kiyoshi Yasuoka, Yutaro Mori, Koji Tsuboi, Hideyuki Sakurai

**Affiliations:** 1 Proton Medical Research Center, University of Tsukuba, 1-1-1 Tennodai, Tsukuba, Ibaraki, 305-8575, Japan; 2 Next Generation Medical Physics Research Program, Research Center for Charged Particle Therapy, National Institute of Radiological Sciences, Inage-ku, Chiba, Japan; 3 Department of Radiological Health Science, Institute of Industrial Ecological Sciences, University of Occupational and Environmental Health, Japan, Kitakyushu, Fukuoka, Japan

**Keywords:** spread-out Bragg peak, proton, oxygen enhancement ratio, relative biological effectiveness

## Abstract

There are few reports on the biological homogeneity within the spread-out Bragg peak (SOBP) of proton beams. Therefore, to evaluate the relative biological effectiveness (RBE) and the oxygen enhancement ratio (OER), human salivary gland tumor (HSG) cells were irradiated at the plateau position (position A) and three different positions within a 6-cm-wide SOBP (position B, 26 mm proximal to the middle; position C, middle; position D, 26 mm distal to the middle) using 155-MeV/n proton beams under both normoxic and hypoxic conditions at the Proton Medical Research Center, University of Tsukuba, Japan. The RBE to the plateau region (RBE_plateau_) and the OER value were calculated from the doses corresponding to 10% survival data. Under the normoxic condition, the RBE_plateau_ was 1.00, 0.99 and 1.09 for positions B, C and D, respectively. Under the hypoxic condition, the RBE_plateau_ was 1.10, 1.06 and 1.12 for positions B, C and D, respectively. The OER was 2.84, 2.60, 2.63 and 2.76 for positions A, B, C and D, respectively. There were no significant differences in either the RBE_plateau_ or the OER between these three positions within the SOBP. In conclusion, biological homogeneity need not necessarily be taken into account for treatment planning for proton beam therapy at the University of Tsukuba.

## INTRODUCTION

Proton beam therapy (PBT) is used to treat various kinds of tumor, and is especially advantageous compared with photon beam therapy for the treatment of large melanomas or deep-seated chordomas [1]. As accelerated proton particles mostly dissipate their energy at the end of the track, thus forming the Bragg peak, the range of the peak is shifted to generate an appropriately sized treatment field (spread-out Bragg peak; SOBP). The biological effect is adjusted to be almost flat, so that the SOBP covers the whole tumor homogeneously.

In clinical settings, the factor of 1.1 has been adopted as the relative biological effectiveness (RBE) for PBT, according to data from numerous biological experiments [2]. A factor ranging from 2.5–3.0 is generally adopted as the oxygen enhancement ratio (OER) for PBT, although the precise data on OER are still limited [3, 4]. However, recent biological experiments have reported that the RBE within the SOBP is not constant; the RBE increases at the distal end of the SOBP [5, 6]. Thus, gradation of the RBE or the OER within the SOBP would need to be considered in planning PBT when the target is exposed from a single direction or the critical structure is located very close to the distal end of the SOBP [2]. Therefore, in this study, we examined the RBE as well as the OER within the SOBP clinically used for PBT at the University of Tsukuba.

## MATERIALS AND METHODS

### Cell culture

Cells originating from a human salivary gland tumor (HSG) cells were used in this study [7], since HSG cells are a standard reference cell line for intercomparison experiments at a range of facilities in Japan [3, 8, 9]. The HSG cells were grown in minimum essential medium (MEM; Sigma-Aldrich, Tokyo, Japan) supplemented with 100 μg/ml streptomycin, 100 U/ml penicillin (Sigma-Aldrich), and 10% heat-inactivated fetal bovine serum. Cells were maintained at 37°C in a humidified incubator with 5% CO_2_ in air, and were harvested with 0.25% trypsin-EDTA in phosphate-buffered saline. Approximately 200 000 cells were then seeded in the central part of 3.8-cm-diameter glass dishes in 200 μl of medium, and cultured for 24 h prior to the exposure.

### Proton beam irradiation

**Fig. 1.**
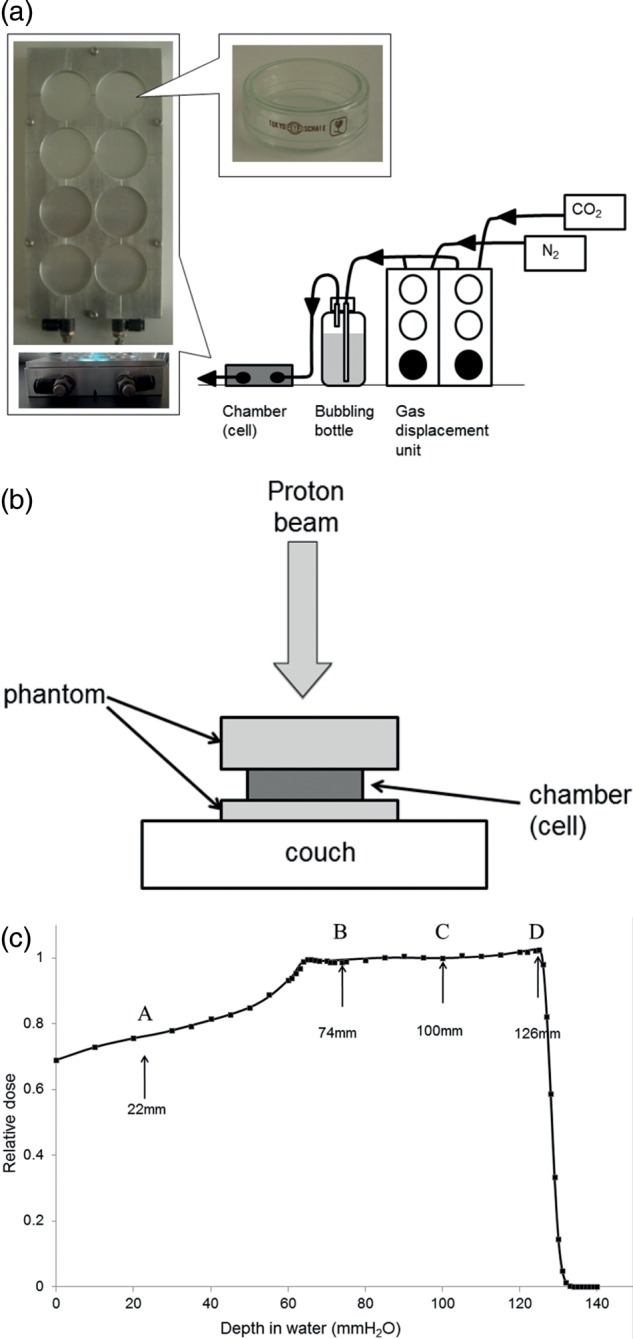
The setup for the sample irradiation (**a** and **b**) and depth–dose distribution of the spread-out Bragg peak (SOBP) of proton beams (**c**). The closed squares represent the relative absorbed dose at various depths in the water-equivalent phantom. The solid line shows a curve fit freehand.

The dishes with cells were filled with 1.2 ml of MEM only and transferred into the irradiation chamber. For irradiation under hypoxic conditions, the chamber was flushed for 1 h before irradiation with 1000 ml/min of a mixture of 95% N_2_ and 5% CO_2_ that had been bubbled through water to maintain high humidity (Fig. 1a). The oxygen concentration was controlled to a partial pressure of <0.2 mmHg, as reported previously [3, 10].

The chamber placed on the couch was irradiated using vertical proton beams (Fig. 1b). A 6-cm-wide SOBP was generated using mono-energetic 155-MeV/n proton beams attenuated by ridge-shaped filters at the Proton Medical Research Center (PMRC) at the University of Tsukuba, Japan. Depth–dose distribution (Fig. 1c) was measured using a silicon diode detector at various depths in the water phantom. The beams were also attenuated by a solid water phantom to adjust the cells to the positions at the plateau (position A in Fig. 1c; 22 mm depth in water), 26 mm proximal to the middle (position B in Fig. 1c; 74 mm depth in water), middle (position C in Fig. 1c; 100 mm depth in water), and 26 mm distal to the middle (position D in Fig. 1c; 126 mm depth in water). The cells were irradiated at 1, 2, 4, 6 and 8 Gy for the normoxic condition, and 2, 5, 8, 14 and 20 Gy for the hypoxic condition.

### Relative biological effectiveness and oxygen enhancement ratio

After irradiation, the cells were seeded in three 6-cm culture dishes and then incubated for ∼13 d. Colonies containing more than 50 cells were scored as survivors. The experiments were replicated three times separately. The surviving fractions were fitted by the linear–quadratic model, as shown by equation (1):

(1) $$\text{S}=\text{exp}(-\alpha \text{D}-\beta {\text{D}}^{2}),$$

where D is the absorbed dose, and α and β are parameters to characterize the cell survival curves.

To investigate the biological flatness of the SOBP, the RBE to the plateau (RBE_plateau_) was calculated from doses corresponding to 10% survival (D_10_) values at the plateau position (D_10, plateau_) and three positions within the SOBP (D_10, SOBP_), as shown by equation (2):

(2) $${\text{RBE}}_{\mathrm{plateau}}={\text{D}}_{10,\;\mathrm{plateau}}/{\text{D}}_{10,\;\mathrm{SOBP}}$$

The OER values in each position were calculated from the ratio of the D_10_ values under normoxic and hypoxic conditions, as shown by equation (3):

(3) $$\text{OER}={\text{D}}_{10,\;\mathrm{hypoxia}}/{\text{D}}_{10,\;\mathrm{normoxia}},$$

where D_10, hypoxia_ and D_10, normoxia_ are, respectively, the doses of protons for 10% survival under hypoxic and normoxic conditions.

### Statistical analysis

Differences were statistically analyzed using a two-sided Mann–Whitney *U*-test. Differences with *P* < 0.05 were considered significant.

## RESULTS AND DISCUSSION

### Relative biological effectiveness within the SOBP of proton beams

**Fig. 2.**
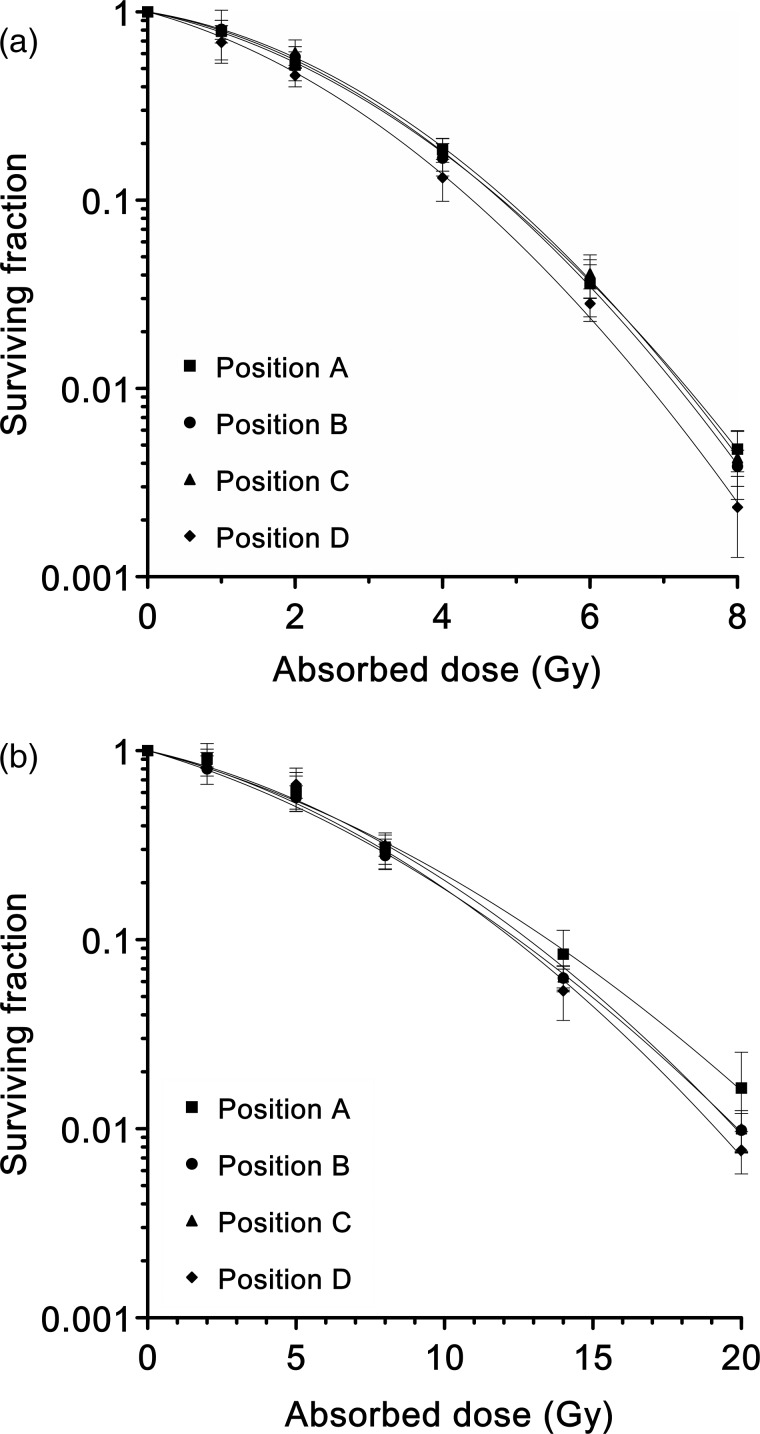
Cell survival curves for each position under normoxic (**a**) and hypoxic (**b**) conditions. Each curve represents the average of three independent experiments fitted by the linear–quadratic model. Error bars indicate the standard deviation.

**Fig. 3.**
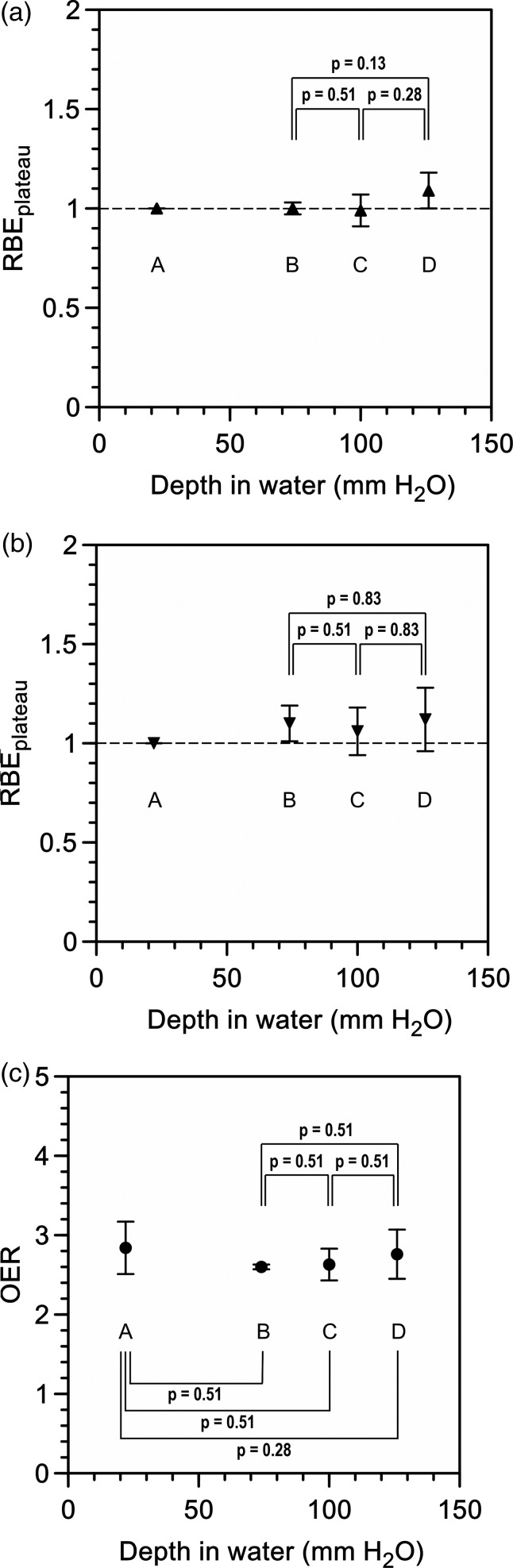
The relative biological effectiveness to the plateau region (RBE_plateau_) under normoxic (**a**) and hypoxic (**b**) conditions, and the oxygen enhancement ratio (OER) (**c**).

Cell survival curves for normoxic and hypoxic conditions at each position within the 6-cm-wide SOBP of the proton beams are shown in Fig. 2. The parameters of the irradiation points and the survival curves (including the D_10_ values and the RBE_plateau_) are summarized in Table 1. No significant differences were demonstrated in the RBE_plateau_ value between any of the positions within the SOBP under either normoxic or hypoxic conditions (Fig. 3a and b).

**Table 1.** Biological flatness within SOBP of proton beam

Irradiated condition	Irradiation position	Depth in water (mm)	α (Gy^−1^)	β (Gy^−2^)	R^2^	D_10_ (Gy)	RBE_plateau_	Irradiation position	OER
normoxic	A	22	0.19 ± 0.02	0.059 ± 0.001	1.00	4.81 ± 0.16	NA	A	2.84 ± 0.33
	B	74	0.17 ± 0.01	0.066 ± 0.003	1.00	4.79 ± 0.06	1.00 ± 0.03	B	2.60 ± 0.03
	C	100	0.15 ± 0.01	0.067 ± 0.005	0.99	4.88 ± 0.22	0.99 ± 0.08	C	2.63 ± 0.20
	D	126	0.24 ± 0.12	0.064 ± 0.018	0.99	4.40 ± 0.23	1.09 ± 0.09	D	2.76 ± 0.31
hypoxic	A	22	0.09 ± 0.00	0.0059 ± 0.0019	0.98	13.64 ± 1.20	NA		
	B	74	0.11 ± 0.00	0.0063 ± 0.0004	0.99	12.45 ± 0.19	1.10 ± 0.09		
	C	100	0.08 ± 0.02	0.0076 ± 0.0007	0.98	12.83 ± 0.39	1.06 ± 0.12		
	D	126	0.10 ± 0.03	0.0076 ± 0.0011	0.96	12.13 ± 0.89	1.12 ± 0.16		

SOBP = spread-out Bragg peak, D_10_ = 10% survival, R^2^ = coefficient of determination, RBE_plateau_ = relative biological effectiveness to the plateau region, OER = oxygen enhancement ratio, NA = not available. Data represents mean ± standard deviation.

A factor ranging from 1.0–1.1 was adopted as the RBE in the SOBP for PBT [2, 8, 11, 12]. However, recent biological experiments showed that the RBE value increases at the distal end of the SOBP, and that there is a larger increase for the fall-off side because of a high linear energy transfer (LET) component of the proton beams just before the terminal of a track [2, 5, 6, 13]. Thus, we should reduce the absorbed dose at the distal end of the SOBP so that the biological effectiveness is flattened in the entire SOBP, especially when a critical organ is close to the target or when the target is exposed from single direction [2]. Our data demonstrated a slight increase in the RBE_plateau_ at the distal end of the SOBP (position D); however, no significant differences were seen between the three positions within the SOBP under either normoxic or hypoxic conditions (Fig. 3a and b). Therefore, the biological effectiveness is almost flat within a 6-cm-wide SOBP for PBT.

### Oxygen enhancement ratio within the SOBP of proton beams

The OERs are shown in Table 1. There were no significant differences in the OER values between any of the positions in this experiment (Fig. 3c).

Although the OER for low-LET photons is reported to be 2.5–3, there are few reports concerning the OER value for proton beams [3, 4]. Wenzl *et al*. reported that the OER value at oxygen levels of 0.5 mmHg was 2.11, 2.08 and 2.04 for the proximal end, middle and distal end of the SOBP, respectively [4]. Our data are consistent with data from their study, and indicate that the OER value for clinical 6-cm-wide SOBP proton beams for PBT is homogenous.

## CONCLUSION

In conclusion, biological parameters such as the RBE_plateau_ and the OER are flat within the SOBP (that is simply adjusted by the absorbed dose); thus there is no need to take into account their homogeneity during treatment planning for PBT at the University of Tsukuba.

## FUNDING

This research is partly supported by institutional sources and in part by Grant-in-Aid (Nos 24390286 and 24300179) from the Ministry of Education, Science, Sports and Culture of Japan. Funding to pay the Open Access publication charges for this article was provided by Grant-in-Aid (No 25670616) from the Ministry of Education, Science, Sports and Culture of Japan.
